# Refining predictors of long-term NEDA-3 status in relapsing-remitting multiple sclerosis: insights from real-world data

**DOI:** 10.1007/s10072-026-08876-x

**Published:** 2026-02-12

**Authors:** Tommaso Guerra, Antonella Bianco, Chiara Esposto, Donata Intini, Fabio Amati, Giuseppe Guglielmini, Francesca Caputo, Damiano Paolicelli, Pietro Iaffaldano

**Affiliations:** 1 Department of Translational Biomedicine and Neurosciences (DiBraiN), University of Bari “Aldo Moro”, Bari, Italy; 2 Ospedale della Murgia “Fabio Perinei”, Neurology Unit, Altamura, Italy; 3 Department of Neurology, SS. Annunziata Hospital, Taranto, Italy

**Keywords:** Multiple sclerosis, Prognostic factors, NEDA-3, DMTs

## Abstract

**Background:**

No evidence of disease activity-3 (NEDA-3) constitutes a crucial target for relapsing-remitting multiple sclerosis (RRMS). Long-term predictors of its attainment remain insufficiently characterized. This study aimed to assess the prevalence and the predictors of long-term NEDA-3 status in a large real-world RRMS cohort.

**Methods:**

This retrospective monocentric study analyzed RRMS patients strictly followed for up to six years. Treatment exposure was classified based on the efficacy of the first therapy in moderate (ME) and high efficacy (HE) disease modifying treatments (DMTs). Percentages of patients exposed to ME and HE DMTs reaching NEDA-3 were compared. Logistic regression models were applied to identify predictors of NEDA-3 achievement at 2 and 5 years. EBNA-1 IgG and VCA IgG from Epstein-Barr virus (EBV) were also analyzed in a small sub-cohort.

**Results:**

485 RRMS patients were included. Subjects starting treatment with HE DMTs were associated with significantly higher rates of NEDA-3 across all time-points. Significant risk factors for not achieving NEDA-3 included multifocal onset, delayed treatment initiation and spinal cord lesions. Conversely, treatment initiation with HE DMT was a significant protective factor for NEDA-3 status achievement at 2 and 5 years of follow-up. Early initiation of HE DMTs significantly improves long-term control of disease activity.

**Conclusions:**

Multifocal onset, delayed treatment initiation, presence of spinal cord lesions and oligoclonal bands were identified as risk factors of not achieving NEDA-3 status.

**Supplementary Information:**

The online version contains supplementary material available at 10.1007/s10072-026-08876-x.

## Introduction

The systematic investigation of clinical prognostic indicators linked to disease activity in multiple sclerosis (MS) is essential in supporting therapeutic choices, even though there is mounting evidence that neurodegenerative processes other than neuroinflammation-related events drive disease progression over time [1]. No evidence of disease activity-3 (NEDA-3) is a composite outcome defined by the absence of clinical relapses, magnetic resonance imaging (MRI) activity, and disability worsening evaluated with changes in Expanded Disability Status Scale (EDSS) scores [2]. The role of NEDA-3 as a reliable indicator of disease activity-free status has been discussed over the last years in real-world MS cohorts [3–5]. Although the number of relapses has decreased and the disability accrual has slowed due to the increasing number of available disease-modifying treatments (DMTs), some patients still experience clinical disease activity and disease progression [6, 7]. A tangled combination of degenerative and inflammatory molecular mechanisms are thought to be responsible for smouldering-associated worsening in MS [8]. Therefore, it can be difficult to predict which patients will attain and maintain NEDA, and the study of clinical and laboratory predictors of its attainment becomes useful in clinical practice. High efficacy (HE) DMTs, especially used in an early phase of the disease, have been demonstrated to be crucial in minimizing and delaying disability accrual in MS [9]. Achieving NEDA is significantly more likely in patients treated with HE DMTs than with moderate efficacy (ME) therapies [10, 11]. The prognostic role of NEDA is still a matter of debate in the MS research field, considering the limitations of this parameter [12]. In this scenario, real-world observational studies represent a great source to explore the prognostic role of NEDA-3 in diverse patient populations [13]. The aim of the study was to analyze the prevalence of NEDA-3 status achievement, assessing also how the timing and type of DMTs influence disease outcomes, and to evaluate clinical, laboratory, radiological, and therapeutic factors associated with its attainment in a large cohort of relapsing remitting MS (RRMS) patients.

## Materials and methods

### Study population

We evaluated the cohort of patients followed at the Multiple Sclerosis Center of the University Hospital Policlinico of Bari.

Patients included in the analysis met the following criteria:(1) RRMS diagnosis according to the 2017 McDonald criteria; (2) first visit within one year of disease onset; (3) at least one neurological visit per year; (4) a minimum of three evaluations; (5) at least two years of follow-up; (6) treatment with at least one DMT; (7) annual MRI follow-up. The patient selection flowchart is shown in Supplementary Fig. 1.

All the data about MS history, demographics, treatments, and regular follow-up of the followed MS patients were extracted from the Italian MS and Related Disorders Register (RISM) [14]. The RISM was approved by the ethical committee at the “Azienda Ospedaliero – Universitaria – Policlinico of Bari” (Study REGISTRO SM001 – approved on 8 July 2016) and by local ethics committees in all participating centers. Patients signed an informed consent to collect and use their clinical data for research purposes. To ensure reliability of results, only patients with complete longitudinal clinical and MRI data fulfilling all inclusion criteria were included in the analysis. Missing baseline variables were integrated through review of local medical records. No imputation methods were applied.

DMTs exposure was classified based on the efficacy of the first prescribed DMT in ME and HE DMTs. The following treatments were classified as ME DMTs: Dimethyl fumarate, Glatiramer Acetate, Interferon-beta products, and Teriflunomide. The following products were considered HE DMTs: Fingolimod, Natalizumab, Ocrelizumab, Cladribine.

NEDA-3 was defined as follows: no relapses, no confirmed EDSS progression from baseline, and no new T2 and/or gadolinium enhancement lesions. A relapse was defined as any new neurological symptom, not associated with infection, lasting for at least 24 h and characterized by new neurological signs. Disability worsening was defined as 1.5-point increase (if baseline EDSS score was 0), 1.0-point increase (if baseline EDSS score was < 5.5), or 0.5-point increase (if baseline EDSS score was 5.5) confirmed 6 months apart. Radiological activity was defined as the occurrence of gadolinium-enhancing lesions or new/enlarged T2-hyperintense lesions. MRI assessment was performed approximately once a year: both brain MRI and MRI of the spinal cord were included in the follow-up assessment, and gadolinium administration was performed at all scans collected. The NEDA status was considered reached only if all the above parameters were fulfilled.

In a subgroup of patients, we retrieved Epstein-Barr virus (EBV) serology, assessing the presence of immunoglobulins (Ig) G antibodies to EBV nuclear antigen 1 (EBNA1) and IgG and IgM to viral capsid antigen (VCA), assessed with chemiluminescent immunoassays (CLIA). Results expressed the concentration of antibodies (U/mL).

### Statistical analysis

Summaries of continuous variables have been calculated as median with interquartile ranges (IQR) or mean and standard deviation (SD) according to the appropriate distribution, and categorical variables have been presented as frequencies (proportions). Percentages of patients exposed to ME and HE DMTs reaching NEDA-3 status at 2, 3, 4, 5 and 6 years of follow-up were compared using the chi-square test.

The association of demographic, clinical, laboratory and radiological baseline factors with the risk of not achieving NEDA-3 status at 2 and 5 years of follow-up were estimated with multivariable logistic regression models, expressed with odds ratios (OR) and 95% confidence intervals (CI). The following covariates were included in the model: age at disease onset, gender (female as reference), type of onset (monofocal/multifocal), presence of IgG oligoclonal bands (OCBs) in the cerebrospinal fluid (CSF), baseline EDSS score, time from onset to the first DMT start, disease duration, first DMT prescribed (classification HE/ME DMTs), presence of spinal cord lesions at baseline MRI. Time from disease onset to first DMT initiation was categorized into quartiles (expressed in years), based on the distribution of the cohort. Possible correlations between EBNA-1 and VCA titers with different clinical variables were also assessed by using Spearman’s rank correlation coefficient.

A p-value of < 0.05 was considered statistically significant. Analyses were performed using Windows IBM SPSS Statistic version 25.

## Results

Clinical data of 5,481 patients were available in the RISM cohort of Bari at the time of data extraction. After applying the inclusion criteria, we retrieved a cohort of 485 RRMS patients, of whom 313 (64.5%) were female. The baseline demographics and clinical characteristics of the overall cohort are detailed in Table 1. The mean (SD) age at onset of 30.65 (11.16) years and the median (IQR) time from diagnosis to DMT initiation was 0.19 (0.08–0.40) years. OCBs were detected in the CSF of 87.9% of patients (*n* = 373) with a median of 14 OCB (0–34) per patient. Mean (SD) follow-up was 7.35 (3.94) years, with a median (IQR) number of neurological evaluations of 9 (3–48) over the observation time. Subjects receiving ME DMTs as first therapy were 351, compared to 134 (27.6%) treated with HE DMTs as first strategy. First DMTs recorded in our cohort are reported in detail in Table 2. Baseline characteristics stratified by initial treatment strategy are reported in Supplementary Table 1. In the HE DMTs group, the majority of patients received fingolimod, 43 (32.09%), and natalizumab, 40 (29.85%). ME DMTs administered more frequently were dimethyl fumarate in 69 (19.49%) subjects and interferon-beta products in 223 (63.85%) patients.

**Table 1:** Baseline clinical and demographic characteristics of the entire cohort

VARIABLE	*N*= 485
Female sex, n (%)	313 (64.5)
Age at onset, mean ± SD, years	30.65 ± 11.16
Time from onset to diagnosis, median (IQR), years	0.41 (0.18-0.86)
Time from diagnosis to first DMT start, median (IQR), years	0.19 (0.08-0.40)
Follow up (mean ± SD), years	7.35 ± 3.94
Age at first DMT start (mean ± SD), years	32.23 ± 11.42
EDSS at onset¸ median (IQR)	2.0 (0-5.0)
Time from onset to first visit, median (IQR), years	0.44 (0.19-0.77)
Presence of oligoclonal bands in CSF, n (%)	373 (77)
Number of oligoclonal bands in CSF, median (IQR)	14 (1-34)
Disease duration (mean ± SD), years	7.63 ± 3.57
Age at the time of the analysis, (mean ± SD), years	39.53 ± 11.68
Patients updated according to RISM, n (%)	432 (89.07)
Number of visits for each patient, median (IQR)	9 (3-48)
Patients starting with HE-DMTs, n (%)	134 (27.6)
Type of MS onset, multifocal n (%)	104 (23)

Abbreviations: *DMTs*, disease-modifying treatments; *HE*, high-efficacy; *ME*, moderate-efficacy; *CSF* cerebrospinal fluid; *RISM* Italian MS and related disorders register

**Table 2:** Patients starting treatment with HE DMTs (A) and ME DMTs (B)

**Patients starting with HE DMTs**	*N*=134
Fingolimod	43 (32.08)
Cladribine	38 (28.35)
Ocrelizumab	13 (9.70)
Natalizumab	40 (29.85)
**Patients starting with ME DMTs**	*N*=351
Teriflunomide	18 (5.12)
Dimethyl fumarate	69 (19.68)
Interferon-beta products	223 (63.53)
Glatiramer acetate	41 (11.68)

Abbreviations: *DMTs*, disease-modifying treatments; *HE*, high-efficacy; *ME,* moderate-efficacy

**Fig. 1:**
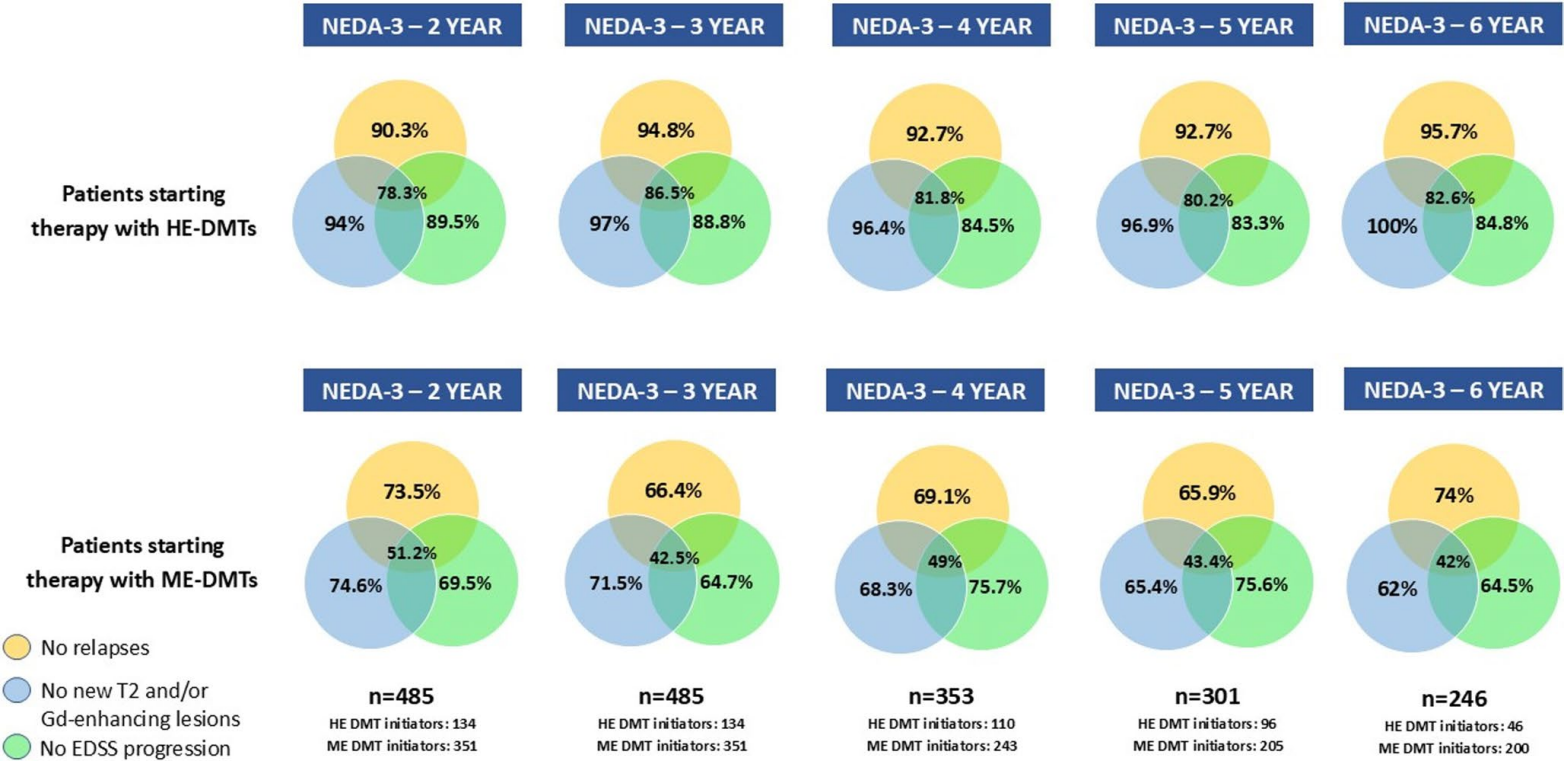
Descriptive overview of the percentages of NEDA-3 achievement over time among patients initiating different high-efficacy disease-modifying therapies (cladribine, fingolimod, natalizumab, and ocrelizumab), stratified by year of follow-up

**Fig. 2:**
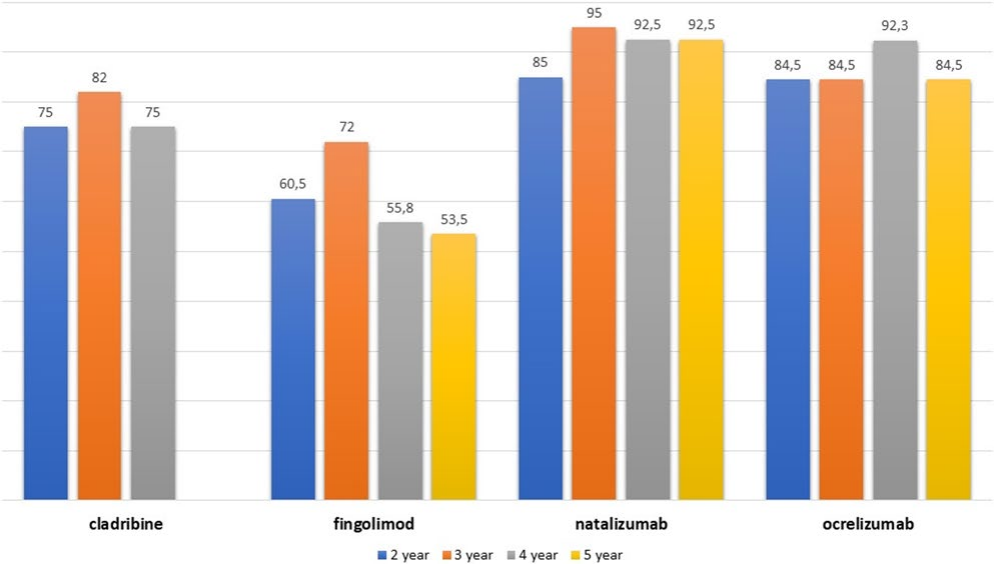
Descriptive overview of the percentages of NEDA-3 achievement and its individual components (no relapses, no new T2 and/or gadolinium-enhancing lesions, and no EDSS progression), stratified by year of follow-up and by initial treatment strategy (high-efficacy vs moderate-efficacy disease-modifying therapies)

The percentage of patients reaching NEDA-3 was assessed yearly from the second to the sixth year, stratified by patients initiating treatment with ME or HE DMTs. Figure 1 shows a descriptive overview of percentages of NEDA-3 achievement and its individual components over time, stratified by initial treatment strategy. Patients who started their treatment history with HE DMTs reached a NEDA-3 status significantly more frequently (*p* = 0.001) than those who received ME DMTs in all the timepoints considered: second year 78.3% vs. 51.2%; third year 86.5% vs. 42.5%, fourth year 81.8% vs. 49%, fifth year 80.2% vs. 43.4% and sixth year 82.6% vs. 42%. The percentage of patients treated with fingolimod as first DMT and reaching NEDA-3 was 60.5%, 72%, 55.8% and 53.5% from the second to the fifth year of follow-up, respectively. Cladribine-treated patients achieving NEDA-3 accounted for 75% in the second year, then 82% and 75% in the third and fourth year. In the ocrelizumab group, the percentages were 84.5%, 84.5%, 92.3% and 84.5% throughout the follow-up time, whereas natalizumab-treated reached NEDA-3 for 85%, 95%, 92.5% and 90.5% from the second to the fifth years of follow-up, respectively. A descriptive overview of NEDA-3 achievement over time across different high-efficacy disease-modifying therapies is shown in Fig. 2. No formal hypothesis testing between treatment groups was performed.

We retrieved the EBV serology of 136 patients. All the subjects were seropositive for EBV at baseline; five patients had low levels (< 20 U/ml) of VCA IgG, in parallel with high levels (> 600 U/ml) of EBNA1 IgG, and therefore could be considered EBV seropositive. Specifically, a level of VCA IgG > 750 U/ml was found in 59 patients, and titers in the range between 10.4 and 750 U/ml for the remaining patients of the cohort. We analyzed the possible correlations between EBNA-1 (> 600 U/ml) and VCA IgG titers with different clinical variables, disease duration, number of relapses in the year before diagnosis and in the two years after, but we did not find significant associations.

Quartiles of time to first DMT initiation were defined according to the cohort distribution and are expressed in years from disease onset: Q1 (≤ 0.47 years, reference group), Q2 (0.48–0.81 years), Q3 (0.82–2.43 years), Q4 (> 2.43 years). The multivariable logistic regression model revealed that a multifocal onset (OR 1.35, 95% CI: 1.15–1.58; 1.53, 1.27–1.79; p-value < 0.001), a longer time to first DMT start (1.46, 1.27–1.69; 1.61, 1.39–1.83; p-value < 0.001) and spinal cord lesions at baseline MRI (1.78, 1.20–2.65; 1.41, 1.05–1.89, p-value 0.004 and 0.002, respectively) were significant risk factors for not achieving NEDA-3 status at 2 and 5 years of follow-up. The presence of IgG OCBs (1.26, 1.12–1.60; p-value 0.005) was a significant risk factor for not achieving NEDA-3 status at 2 years of follow-up. Conversely, treatment initiation with HE DMT (0.79, 0.68–0.90; 0.52, 0.32–0.85; p-value < 0.001) was a significant protective factor for NEDA-3 status achievement at 2 and 5 years of follow-up. (Table 3)

**Table 3:** Multivariable logistic regression model performed on the entire cohort to estimate the predictors of not achieving NEDA-3 status

VARIABLES	2 follow-up year		5 follow-up year	
	OR (95% CI)	*P*-value	OR (95% CI)	*P*-value
Female sex	1.32 (0.68-2.62)	0.406	1.38 (0.69-2.75)	0.345
Age at disease onset	0.63 (0.31-1.29)	0.124	0.74 (0.45-1.96)	0.610
Disease duration	1.28 (0.86–1.90)	0.215	1.11 (0.84–1.42)	0.634
EDSS at onset	1.36 (0.70-2.63)	0.371	1.34 (0.92-1.63)	0.087
Type of clinical onset, monofocal reference group, Multifocal	1.35 (1.15-1.58)	< 0.001	1.53 (1.27-1.79)	< 0.001
Quartile of time to first DMT start, 1° quartile (Q1) reference group, years				
Q4 (>2.43)	1.46 (1.27-1.69)	< 0.001	1.61 (1.39-1.83)	< 0.001
Q3 (0.82-2.43)	1.10 (0.93-1.29)	0.298	1.24 (0.79-1.65)	0.321
Q2 (0.48-0.81)	0.93 (0.75-1.16)	0.450	0.93 (0.70-1.18)	0.670
Presence of spinal cord lesions at baseline MRI	1.78 (1.20-2.65)	0.004	1.41 (1.05-1.89)	0.002
Presence of IgG OCBs n CSF	1.26 (1.12-1.60)	0.005	1.10 (0.75-1.63)	0.347
DMT HE vs. ME DMTs	0.79 (0.68-0.90)	< 0.001	0.52 (0.32-0.85)	< 0.001

Abbreviations: *CSF*, cerebrospinal fluid; *DMTs*, disease-modifying treatments; *EDSS*, Expanded Disability Status Scale; *HE*, high-efficacy; *ME*, moderate-efficacy; *OCB*, immunoglobulin, *Ig*; oligoclonal bands

## Discussion

In this retrospective single-center study, we evaluated the achievement of NEDA-3 status in a large cohort of MS patients treated with different DMTs and followed for up to six years, investigating baseline clinical and laboratory predictors of NEDA-3 status achievement and highlighting the role of treatment strategies. Several authors have explored how NEDA-3 could be a trustworthy biomarker of disease activity-free status [15–17]. A recent review highlighted the role of NEDA as a clinical tool from both the clinician and the patient perspective in everyday MS management and therapeutic decision-making process [18]. Even considering its limitations, this composite outcome allows for a combination of clinical and radiological parameters faced daily by neurologists under a single definition.

In our study a treatment strategy with HE DMTs as first therapy was significantly more effective in achieving NEDA-3 compared to ME DMTs across all time points (e.g., year 2: 78.3% vs. 51.2%, *p* < 0.001). According to a recent study by Signoriello et al., a significant proportion of patients attained NEDA-3 when beginning HE DMT with monoclonal antibodies, without differences between natalizumab and ocrelizumab, in a cohort of highly active MS patients [11]. Early high-efficacy strategy led patients to reach NEDA-3 more frequently, especially in naïve patients [19], , with a superior efficacy consistently seen also in maintaining NEDA status in all epochs of analysis [20]. 

A further comparison between different HE treatments in our study revealed that patients initiating with fingolimod reached less frequently NEDA-3 status compared to natalizumab, ocrelizumab and cladribine-treated patients. A previous observational real-world study already described how different therapeutic alternatives result in different NEDA-3 achievements, with natalizumab resulting superior to fingolimod in RRMS in obtaining NEDA-3 status in the second and fourth year of follow-up [21]. Similar to these results, in years two through five of a Czech research, the percentage of patients treated with natalizumab who achieved NEDA-3 rose to over 70% [22]. A recent study profiled a reduction in relapses and MRI activity, along with a stabilization of disability, in patients treated with cladribine, resulting in 74.9% NEDA-3 at 24 months [23]. Conversely, among patients receiving fingolimod, only about half achieved NEDA-3 status in a multi-center, observational longitudinal cohort study [24]. 

The identification of inflammatory and neurodegenerative biomarkers may help identify a series of cellular and molecular processes that take place both inside and outside the central nervous system, going beyond clinical NEDA [25, 26]. 

The multivariable logistic regression model showed that treatment initiation with HE DMT was a significant protective factor for achieving NEDA-3 status at 2 and 5 years of follow-up. Conversely, a multifocal onset, a longer delay to the first DMT start, the presence of OCBs and spinal cord lesions at baseline MRI scan were identified as significant risk factors for not achieving NEDA-3 status at those time points. A precocious initiation of DMTs early in the disease course is beneficial for minimizing the disability accrual [9], and a delayed DMT initiation was associated with a higher risk of progression independent of disease activity (PIRA) and relapses-associated worsening events in a large cohort of MS patients from RISM [27]. HE therapies commenced within 2 years of disease onset resulted associated with less disability after 6–10 years than when commenced later in the disease course [28]. The multivariable model included in the analysis led us to thoughtful consideration about prognostic factors of the MS course. The prognostic role of spinal cord lesions at onset has been already assessed in several studies, outlining a consistent association with EDSS worsening and SPMS conversion at 15 years [29], alongside the occurrence of PIRA events [3]. A multifocal onset was associated also with a higher risk of SPMS conversion [30]. The presence of OCB in CSF is considered a milestone in the diagnosis of MS. While it is necessary to state that the number of OCBs is related in some studies to long-term prognosis, this topic is constantly evolving in the scientific community as new immunological biomarkers become available [31]. 

The retrospective design of our study did not allow us to consider EBV serology for all patients: EBV immunology was available for patients diagnosed after hospitalization or day service procedures at our MS centre, and not available for the remaining patients, followed at our center but not diagnosed here or with missing data in the baseline tests. EBV infection is thought to be the biggest risk factor for MS development. Increased MS risk is linked to particularly high levels of IgG against EBNA1, with a proposed molecular mimicry mechanism in which these antibodies to EBNA1 interact with CNS proteins like GlialCAM, alpha-crystallin B, and anoctamin 2 [32–34]. A recent study found a negative correlation between EBNA-1 IgG titers and the number of relapses two years before starting DMTs [35]. A triggering correlation between EBNA-1 IgG titers and PIRA was found in another study, highlighting decreased levels in 20.0% of PIRA patients and 73.3% of subjects with relapse-associated worsening [36]. In a pediatric-onset MS cohort, the negativity for anti-EBV IgG antibodies at diagnosis was associated with higher achievement of NEDA-3 status at 12 months [37]. 

Several limitations of this study deserve discussion. We were unable to provide more insights into MRI parameters, due to the lack of standardized radiological protocols in our center, and we evaluated MRI activity considering solely the presence or absence of new lesions or gadolinium enhancement. The collection of data was retrospectively performed considering the extraction of data from RISM-App and paper medical records, thus lacking the power of a prospective collection performed ad hoc on the cohort. Disability accrual is currently known to be caused by both relapse-related and PIRA events over time, and NEDA-3 may not guarantee long-term clinical stability, not capturing properly phenomena of silent progression. The prognostic relevance of first choices in terms of therapeutic management and evaluation of baseline clinical, demographic, and radiological data at disease onset and diagnosis was the final focus of our study. Therefore, a further major limitation of our analysis is that patients were classified according to the first DMT received, without accounting for subsequent treatment switches during follow-up. This may have introduced bias in the estimation of long-term NEDA-3 outcomes, as treatment changes could have influenced disease activity independently of baseline predictors. Propensity score–based methods were not applied in this study due to the relatively limited sample size of the HE DMTs cohort, the risk of substantial loss of statistical power after matching, and the presence of unmeasured clinical factors influencing treatment strategy that are only partially captured by baseline variables. As expected in a real-world observational setting, we acknowledge the risk of indication bias. Time-to-event analyses were not performed due to the focus on predefined clinically relevant time points and the heterogeneity of follow-up intervals and clinical assessments in a retrospective real-world setting. In addition, it was not possible to assess the antibody titer against EBV during treatment with DMT, given the retrospective nature of the study. Despite these considerations, for neurologists treating RRMS patients NEDA-3 is a pertinent treatment target in real clinical practice. Since the study of NEDA-3 for more than two years from a DMT start has only been documented in a small number of studies, it is crucial to investigate what happens to this measure over time [38]. 

In conclusion, our findings reinforce the value of NEDA-3 as a clinical treatment target in RRMS and underscore the superior efficacy of HE DMTs in achieving and sustaining this outcome. Higher disease activity, spinal cord lesions, OCBs in CSF and a delayed treatment start were confirmed risk factors for not achieving NEDA-3 status. Future research should incorporate broader biomarkers of disease progression, including PIRA to enhance the predictive value of composite measures in MS.

## Supplementary Information

Below is the link to the electronic supplementary material.

Supplementary Material 1

## Data Availability

Anonymized data will be shared on reasonable request from a qualified investigator.
