# Supplementary material for: Refining predictors of long-term NEDA-3 status in relapsing-remitting multiple sclerosis: insights from real-world data
Source: Neurol Sci. 2026 Feb 12;47(3):251. doi: 10.1007/s10072-026-08876-x (PMC12894112; doi:10.1007/s10072-026-08876-x)
Supplement: Supplementary file 1 — Supplementary Material 1 [file 10072_2026_8876_MOESM1_ESM.docx]

**Supplementary materials**


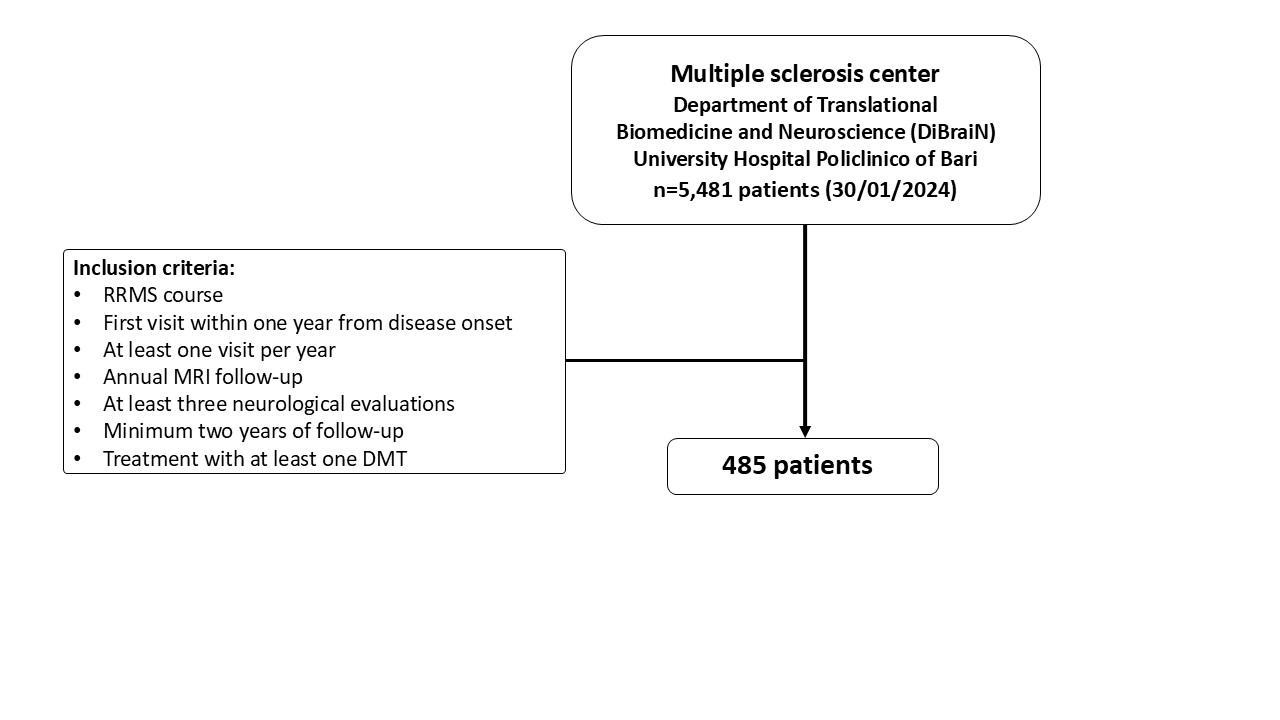


**Supplementary Figure 1. Flowchart of patients’ selection procedure.**

**Supplementary Table 1. Baseline clinical characteristics stratified by initial treatment strategy.**

|  | **Overall**  **(485)** | **Patients first treated with ME DMTs**  **(351)** | **Patients first treated with HE DMTs**  **(134)** | **SMD** |
| --- | --- | --- | --- | --- |
| **Female sex, n (%)** | 313 (64.5) | 223 (63.5) | 90 (67.2) | 0.08 |
| **Age at onset, mean ± SD, years** | 30.65 ± 11.16 | 30.84 ± 10.87 | 30.16 ± 11.91 | -0.06 |
| **Time from onset to diagnosis, median (IQR), years** | 0.41 (0.18-0.86) | 0.36 (0.16-0.68) | 0.78 (0.25-1.21) | 0.10 |
| **Time from diagnosis to first DMT start, median (IQR), years** | 0.19 (0.08-0.40) | 0.18 (0.07-0.36) | 0.24 (0.11-0.50) | 0.11 |
| **Follow up (mean ± SD), years** | 7.35 ± 3.94 | 9.34 ± 3.30 | 6.68 ± 2.85 | -0.84 |
| **Age at first DMT start (mean ± SD), years** | 32.23 ± 11.42 | 31.73 ± 11.0 | 33.53 ± 12.41 | 0.15 |
| **EDSS at onset¸ median (IQR)** | 2.0 (0-5.0) | 2.0 (0-5.0) | 2.5 (1.0-5.0) |  |
| **Presence of oligoclonal bands in CSF, n (%)** | 373 (77) | 256 (75.21) | 109 (81.34) |  |
| **Disease duration (mean ± SD), years** | 7.63 ± 3.57 | 8.65 ± 3.30 | 4.98 ± 2.85 | 1.15 |
| **Number of visits for each patient, median (IQR)** | 9 (3-48) | 10 (7-16) | 6 (7-16) | 0.08 |
| **Type of MS onset, multifocal n (%)** | 104 (23) | 69 (19.66) | 35 (26.12) |  |

**Continuous variables were summarized as mean (SD) or median (IQR) according to their distribution. Standardized mean differences were calculated on continuous variables regardless of the summary statistic displayed.*

Abbreviations: disease-modifying treatments, DMTs; high-efficacy, HE; moderate-efficacy, ME; cerebrospinal fluid, CSF; Italian MS and related disorders Register, RISM; standardized mean differences, SMD.
